# Vocal behaviour of allied male dolphins during cooperative mate guarding

**DOI:** 10.1007/s10071-019-01290-1

**Published:** 2019-07-17

**Authors:** Stephanie L. King, Simon J. Allen, Michael Krützen, Richard C. Connor

**Affiliations:** 1 School of Biological Sciences, University of Bristol, Bristol, BS8 1TQ UK; 2 School of Biological Sciences, University of Western Australia, Crawley, WA 6009 Australia; 3 Evolutionary Genetics Group, Department of Anthropology, University of Zurich, 8057 Zurich, CH Switzerland; 4 Biology Department, University of Massachusetts Dartmouth, North Dartmouth, MA 02747 USA

**Keywords:** Alliance, Bottlenose dolphin, Coalition, Communication, Cooperation, Coordination

## Abstract

Coercive mate guarding, where males use aggression to control female movements, is a form of sexual coercion which functions to constrain female mate choice. Non-human primates, for example, herd females to keep them away from competing males, but male bottlenose dolphins (*Tursiops aduncus*) also herd females to keep them close to their alliance partners. Indeed, pairs and trios of male dolphins work together to sequester single estrus females and defend them from competing alliances. Yet how males facilitate such coordination remains unknown. Here, we investigate the vocal behaviour of allied male bottlenose dolphins during the herding of individual females, examining how the production of whistles and ‘pops’ (a threat vocalisation) varied with behavioural state and inter-animal distances. Allied males produced both whistles and pops significantly more often and at higher rates during social interactions, though they differed in function. Whistle rates increased significantly when new individuals joined the consorting group, consistent with previous work showing that whistles are part of a greeting sequence for this species. Whistle matching also appeared to play a role in within-alliance coordination. Pop vocalisations increased significantly when the nearest male to the female changed, likely inducing the female to remain close as the males coordinate a guard switch. Building upon prior research examining female movements in response to pops, we show that males approach the female and current guard whilst popping, leading to a guard switch. Our results provide new insights into the use of vocal signals during cooperative mate guarding between allied male dolphins.

**Electronic supplementary material:**

The online version of this article (10.1007/s10071-019-01290-1) contains supplementary material, which is available to authorized users.

## Introduction

Mate guarding is widespread in the animal kingdom and is a significant determinant of male reproductive success (Girard-Buttoz et al. 2014). It encompasses a number of behavioural strategies, including monopolising access to female groups (Packer and Pusey 1982) and contesting access to individual females (Birkhead 1979). The guarding of single estrus females by individual males, whereby one male prevents others from securing paternities, is prevalent in both birds and mammals (e.g. magpies, Birkhead 1979; tits, Kempenaers et al. 1995; warblers, Komdeur 2001; elephants Poole 1989; and various non-human primates, Alberts et al. 1996; Van Belle et al. 2009; Girard-Buttoz et al. 2014). The formation of male coalitions and alliances, where multiple males monopolise access to females, is predominant in mammals (Olson and Blumstein 2009; Díaz-Muñoz et al. 2014), such as lions (Packer et al. 1991), Camargue horses (Feh 1999), bottlenose dolphins (Connor and Krützen 2015), chimpanzees (Watts 1998), and Guinea baboons (Patzelt et al. 2014). In most of these cases, males work together to guard groups of females; the cooperative guarding of single females by multiple males is unusual, presumably because female fertilisation is indivisible (Díaz-Muñoz et al. 2014). In fact, such reproductive cooperation, where males form coalitions or alliances to gain or defend access to an indivisible resource (e.g. conceptions) is an evolutionary hurdle relatively few species have overcome (Allen et al. 2017).

In terms of the mechanisms underlying mate guarding, female-directed aggression by males may be used to constrain female movement and mate choice (Muller and Wrangham 2009). Coercive mate guarding is found, for example, in chimpanzees (Muller et al. 2007, 2011), hamadryas and chacma baboons (Kummer 1968; Baniel et al. 2017), bottlenose dolphins (Connor and Krützen 2015), and humans (Flinn 1988). All of these examples of coercive mate guarding are performed by single males, with the exception of bottlenose dolphins, in which ‘consortships’ of single females are initiated and maintained by multiple males working together (Connor and Krützen 2015).

In Shark Bay, Western Australia, Indo-Pacific bottlenose dolphins (*Tursiops aduncus*) exhibit fission–fusion grouping dynamics with strongly differentiated relationships, including nested male alliances (Randić et al. 2012). Male dolphins in this population form long-lasting, cooperative alliances, where the core unit of male social organisation is the second-order alliance, typically comprising 4–14 males (Connor and Krützen 2015). These males engage in coordinated efforts to gain a reproductive advantage over their competitors, typically males belonging to other second-order alliances. Within these second-order alliances, pairs or trios of allied males, known as first-order alliances, work together to herd single estrus females during events termed ‘consortships’ (Connor and Krützen 2015). Multiple first-order alliances from the same second-order alliance may participate in attempts to steal females from competing alliances, or defend against such attempts (Connor and Krützen 2015). These strong alliance relationships can last for decades and are critical to each male’s reproductive success (Connor and Krützen 2015); males cannot monopolise and defend females on their own due to the intense competition for receptive females, minimal sexual size dimorphism in the species, and the three-dimensional habitat that impedes coerced matings (Connor et al. 2000, 2017). It remains unknown as to how allied males facilitate such coordination, and whether males communicate acoustically in coordinating their cooperative efforts.

We investigated the role of vocal signals between allied male dolphins during coercive mate guarding in Shark Bay. Bottlenose dolphins possess a flexible communication system due to their propensity for vocal production learning, a notably rare skill in mammals (Janik 2014). Allied male dolphins in this population use signature whistles to broadcast individual identity (King et al. 2018). During consortships, they frequently use a purported threat vocalisation called ‘pops’ (Connor and Smolker 1996; Vollmer et al. 2015). Pops are narrow-band, low frequency pulses produced in sequential trains of 3–30 pops at rates of 6–12 pops/s (Connor and Smolker 1996). Prior research suggested that pops were a threat vocalisation based on female response to in-air pops produced by three provisioned male dolphins and a strong association with physical threats (Connor and Smolker 1996) and the finding that pops are more likely to be produced when herding females than in other contexts (Vollmer et al. 2015). Here, we conducted focal follows of free-ranging dolphin groups taking behavioural observations and acoustic recordings to explore how allied male dolphins use these two types of vocalisations—pops and whistles—to coordinate behaviour when cooperating in the coercion of females. We also explored how behavioural state and inter-animal distances influence call use by male dolphins during cooperative mate guarding.

## Materials and methods

### Field site

Our long-term dolphin research has been run on a seasonal basis (typically austral winter–spring) since 1982 off Monkey Mia (in the eastern gulf of Shark Bay) and 2007 off Useless Loop (in the western gulf of Shark Bay). Data for this study were collected during 25 focal follows (Altmann 1974) of first-order allied male dolphins and their female consort from a small (5.4 m) research vessel in May–June 2016 in Shark Bay’s western gulf, and August–September 2016 and June–September 2017 in the eastern gulf.

### Behavioural data

We analysed focal follows of first-order male alliances herding a female. Herding is defined as an aggressively maintained association, where two to three males use vocal and physical threats to coerce a female to accompany them. Males engage in normal daily activities, such as foraging, travelling and resting, while herding a female, as well as in social and sexual behaviours directed at the female (e.g. synchronous displays, Connor and Krützen 2015). Individual dolphins in this population are well marked and thus were identified by trained observers on the research vessel via their unique dorsal fin shape and scars. Individual identification was corroborated with photo-identification data collected using a Canon 50D camera and 100–400 mm IS lens. During each focal follow, we verified the following variables at every 5-min interval: group membership and size, as defined by the 10 m ‘chain rule’ (Smolker et al. 1992); predominant group behavioural state (rest, travel, forage and socialise—see ESM for definitions); and predominant group spread, which was visually estimated and classified as tight (inter-animal distance < 2 m or < 1 body length distance (BLD)), moderate (inter-animal distance 2–5 m or 1–2 BLD), spread (inter-animal distance 5–10 m or 2–5 BLD) or widespread (inter-animal distance > 10 m or > 5 BLD). All changes in group membership (e.g. arrivals and departures of individuals) were recorded when they occurred during focal follows. In 2017, additional data were collected on which male was closest to the female during each 5 min observation period. Distance measures used were the same as used for group spread, e.g. if a trio of males are widespread (> 10 m apart) but one of them is tight (< 2 m) with the female, then the predominant group spread is widespread but the closest male is tight. The closest male to the female is considered to be the guard. We systematically recorded predominant closest male to the female every 5-min and also recorded all cases of guard switches when they occurred during focal follows.

### Acoustic analysis

Our hydrophone array consisted of four HTI-96 MIN series (flat frequency response: 0.002–30 kHz ± 1 dB) towed at 1 m depth around our research vessel in a rectangular formation (ca. 2 × 3.5 m), as per that outlined in King et al. (2018). Recordings were made onto a TASCAM DR-680 MKII multi-track recorder at a sampling rate of 96 kHz. A spoken track was used to note the bearing (compass bearing, where the boat’s bow was 0°), distance (m) and identification of the focal individuals at each surfacing. Focal follows were synchronised with the acoustic recording at the start of each follow. All recordings used in the analysis were made when the engine was switched off.

Acoustic recordings were analysed by inspecting the spectrograms (FFT length 1024, Hamming window) in Adobe Audition CC 2017. All occurrences of whistles and pops were identified and visually graded based on their signal-to-noise ratio (1: signal is faint but visible on the spectrogram, 2: signal is clear and unambiguous, 3: signal is prominent and dominates; Kriesell et al. 2014). Frequency-modulated whistles were identified as either continuous in their frequency contour pattern or multi-looped whistles. Multi-loop whistles were defined as a repeated modulation pattern that could be separated by periods of stereotyped silence up to 250 ms in length (Esch et al. 2009).

All vocalisations graded 2 and 3 were included in the analysis. In order to demonstrate that the vocalisations used in this analysis reliably came from our focal group (and, where possible, to identify which group member was vocalising), we localised a subset of whistles and pops (Table S1).

Only calls with good signal-to-noise ratios were used for the localisation analysis. Localisation accuracy of the array was calculated using custom-written MATLAB routines to calculate 2D averaged MINNA (minimum number of receiver array) localisations using the methods described in Wahlberg et al. (2001) and Schulz et al. (2006). The array was calibrated using two different frequency-modulated dolphin whistles, each approximately 1.5 s in duration with a frequency range of 4–20 kHz, as well as two different pop trains previously recorded from this population. Acoustic localisation accuracy for whistle directions (*n* = 75) were calculated as 76% within ± 15**°** of the true location, and 99% within ± 30**°**. Localisation accuracy for pop directions (*n* = 50) were calculated as 68% within ± 5°, 94% within ± 10°, and 100% within ± 15° of the true location. However, variation in estimated direction within a train was low, with < 2**°** difference between sequential pops in the same train.

### Statistical analysis

All statistical procedures were conducted in R (R Core Team 2018). We summed the number of each vocalisation type (whistles and pop trains) in each 5-min observation period and modelled them against behavioural state and group spread (as factors). Any 5-min periods in which group membership was unknown were removed from the analysis (*n* = 5). Our data were both highly zero-inflated, due to the fact that dolphins can be silent for extended periods of time (Table S1), and temporally correlated, due to the nature of focal follows. Thus, to account for temporal correlation in call production (e.g. bouts), we used Generalised Estimating Equations (GEEs) using the *geepack* package in R (R Core Team 2018). We built models with an autoregressive correlation structure, where the focal follow number was the blocking unit, so that calls were correlated within each focal follow but were independent between follows. Zero-inflation can be addressed with zero-altered or hurdle models that are partitioned into two parts (a binary process that models zero and positive counts; and a zero-truncated Poisson process that models only positive counts; Zuur et al. 2009), but these models do not account for temporal correlation. The hurdle model, however, can be carried out manually using binomial and Poisson generalised linear models, which provide the same results in terms of estimated parameters and standard errors (Zuur et al. 2009). We therefore built two types of GEE for each vocalisation type: (1) a call occurrence model to identify how behavioural state and group spread influence the occurrence of a call type, considering binomial distribution to evaluate the presence or absence of calls per observation period; and (2) a call frequency model to identify how behavioural state and group spread affect the frequency of calls when they occur, considering Poisson distribution to model the positive counts of each vocalisation type per observation period. For the frequency model, the logarithm of the number of animals per observation period was included as an offset to account for differences in group sizes when additional individuals joined or left the focal group. We selected the most parsimonious model with the Quasi-likelihood Information Criterion (QIC; Pan 2001) using the *MuMIn* package in R (Bartoń 2009) and sequential Wald tests (anova function in R). Where ΔQIC < 4 between the best models, we used model averaging on the top set of models (Grueber et al. 2011). All models are presented in Table S2.

To explore the relationship between communication and coordinated behaviour in more detail, we built two Generalised Linear Mixed Models with binomial family. In the first model, the response variable was ‘arrival’ (0 = no, and 1 = yes), defined as a new individual(s) joining the focal group during that 5-min period (*n* = 620 across 16 first-order alliances). In the second, we used a subset of data from 2017 where information was available on the closest consorting male to the female (*n* = 194 across 11 first-order alliances), where the response variable was ‘change in closest male to female’ (0 = no change, and 1 = change). For both models, to control for repeated measures of individuals, we set first-order alliance as a random effect. Predictor variables for both models were pop train rate (number of pop trains/group size) and whistle rate (number of whistles/group size). We selected models using Akaike Information Criterion (AIC) and sequential Wald tests (anova function in R) and, where ΔAIC < 4 between the best models, then model averaging was carried out on the top set of models (Grueber et al. 2011). All models are presented in Table S3 and Table S4.

## Results

We collected 52 h of data from 25 focal follows of 16 first-order alliances; comprising 35 individual males and 16 consorted females (Table S1). This resulted in 620 5-min periods used in our analyses. Due to the fission–fusion dynamics of the species, 226 of those 5-min periods included additional animals that temporarily joined our focal group (148 where males from the same second-order alliance joined, sometimes with their own female consort (36%); 71 where males from a different second-order joined, sometimes with their own female consort (24%); and 7 where adult females joined). A total of 1268 whistles and 1221 pop trains were recorded and used in our analyses and, of these, 210 whistles and 293 pop trains were localised to our focal group and/or focal individuals, allowing us to explore calling behaviour in more detail.

**Fig. 1:**
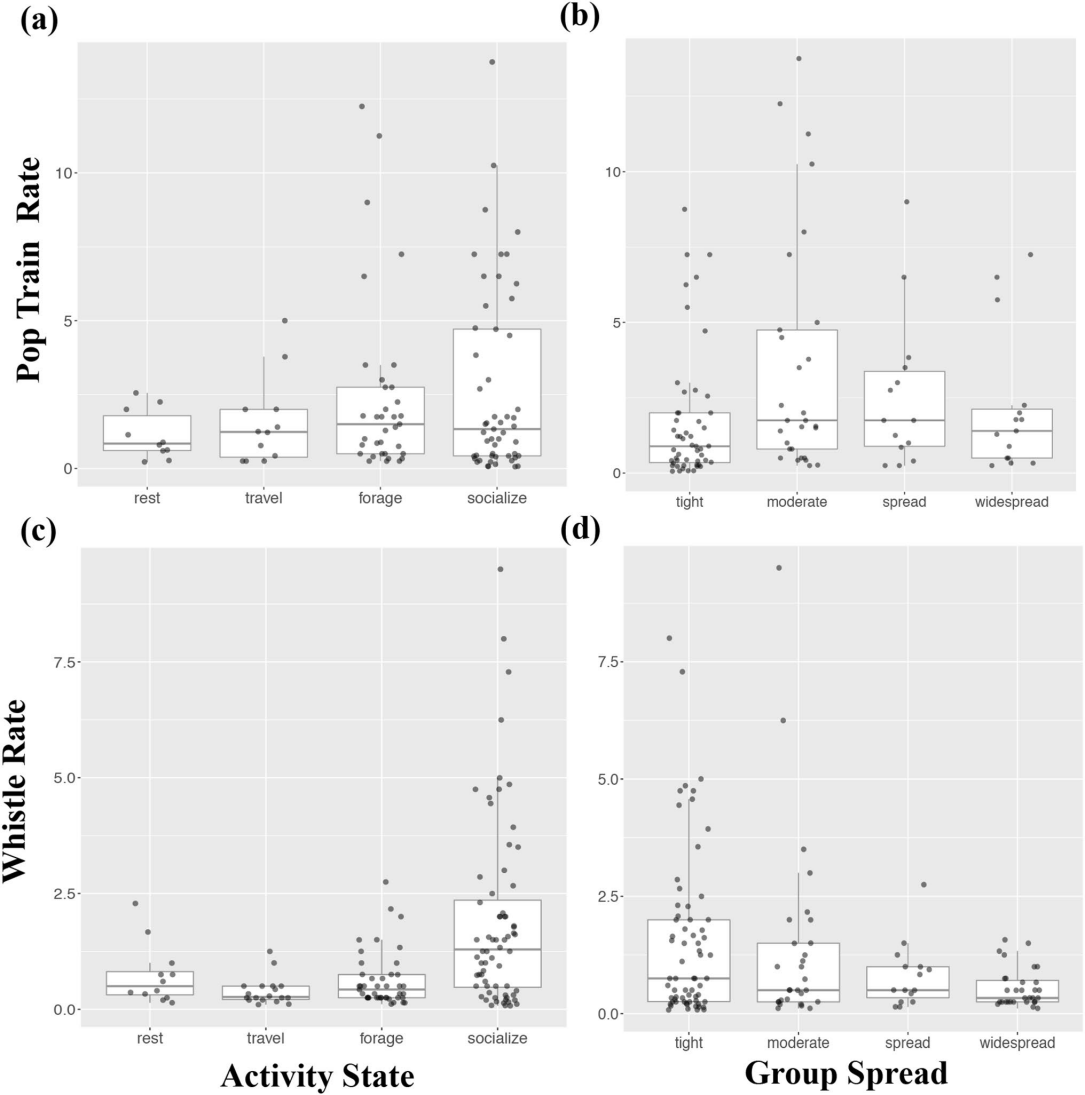
Vocalisation rates as a function of predominant group activity and predominant group spread: panels **a** and **b** show boxplots of non-zero pop train rates, and panels **c** and **d** show boxplots of non-zero whistle rates

**Fig. 2:**
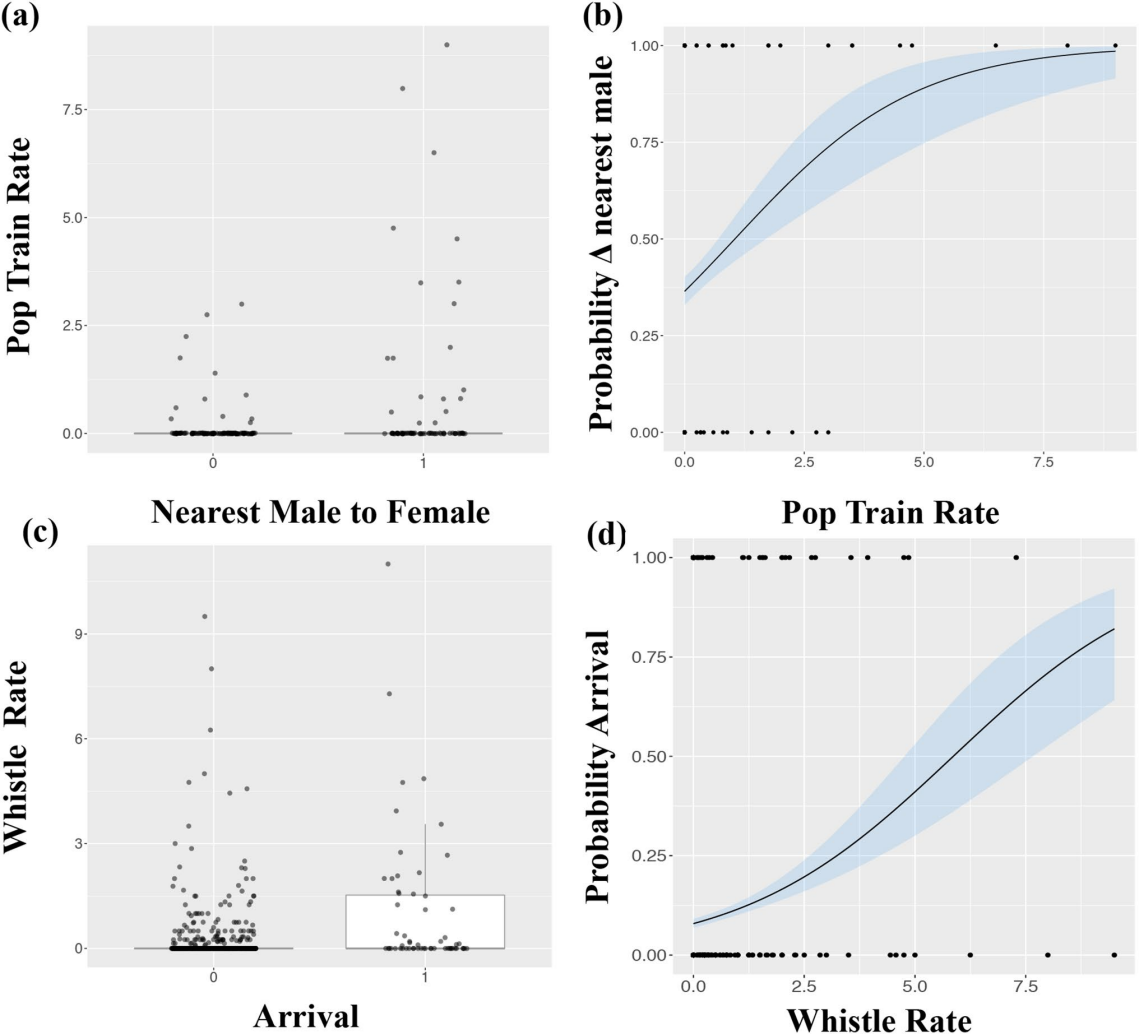
Relationship between vocal behaviour and behavioural coordination: **a** boxplots showing number of pop trains for change in nearest male to female; **b** binomial model predictions for the significant relationship between change in nearest male to female and pop train rate; **c** boxplots showing number of whistles produced for arrival of new individual(s); **d** binomial model predictions for the significant relationship between arrival and whistle rate. Shaded areas represent 95% confidence intervals

**Fig. 3:**
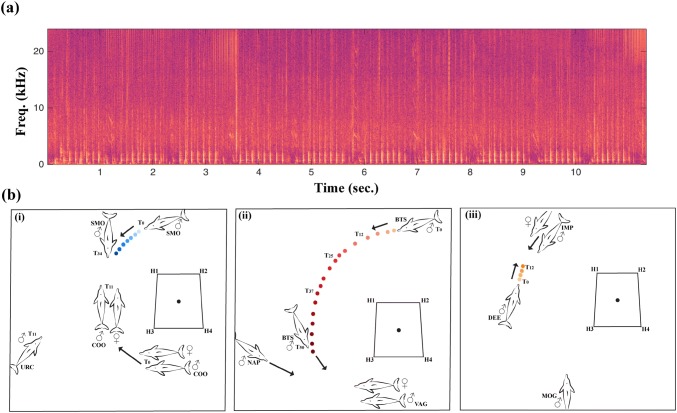
**a** Spectrogram of a pop train sequence produced by an adult male in Shark Bay (down-sampled to 48 kHz, FFT length: 1024, Hanning window function). **b** Examples of pop production prior to guard switches: locations of the consorting males (♂) and female (♀), their individual movement patterns (arrows) in response to localised pop trains (coloured dots) over time (T_i_). The three-letter codes represent animal identification, the hydrophone locations are H1–H4, and the black dot denotes the centre of the array. Panel (1) shows an instance where the popping male then jointly guarded (SMO + COO) the female, and panels (2) and (3) are instances when there was a complete guard switch (VAG to NAP; IMP to DEE). In panel (2) we were unable to determine who was popping once BTS joined with NAP

Overall, pop trains occurred more often and at higher rates when dolphins were socialising, but group spread had no effect on pop production (Table 1, Fig. 1a, b). Pops were not recorded in eight of the 25 follows (Table S1), but during six out of these eight follows the groups were resting and in close proximity (< 2 m) to each other. Interestingly, when the nearest male to female changed (*n* = 78 occurrences across nine first-order alliances) there was a significant increase in pop train rate (Table 2, Fig. 2a, b) but not in whistle rate (Table 2). Under our definition, a change in nearest male to female also included those instances in which males joined the current closest male (resulting in more than one guard), so there was not always a complete guard switch. However, taking a more conservative approach and including only instances in which there was a complete guard switch—i.e. when the incoming male becomes closer to the female than the current closest male (*n* = 27 across nine first-order alliances), the confidence intervals narrowed (glmer: *z* = 3.10, *P* = 0.002, confidence intervals: 0.18-0.81). To investigate this behaviour in finer detail, we localised sequential pop trains to individual males during three different guard switches (Fig. 3) to determine how pops were being used in this context. The localisation data suggest it is not necessarily the nearest male to the female that produces the pops, nor is it always the female that approaches the popping male (Connor and Smolker 1996 examined only female movements in response to pops). In all three cases where pops were localised during a guard switch, the males that approached the female started popping first, even though another male was guarding the female, leading to a guard switch (Fig. 3).

**Table 1:** Parameter estimates for a binomial Generalised Estimating Equation (GEE) model (occurrence) and Poisson GEE (frequency) for pop train and whistle counts as a function of group activity (Travel, Forage, Socialising) and group spread (Tight, Moderate, Spread) categories. Baseline level for activity is ‘Rest’ and for spread is ‘Widespread’

Parameter	Pop train occurrence model				Pop train frequency model			
	Estimate	Std. error	*z*	*P* value	Estimate	Std. error	Wald	*P* value
Intercept	− 2.262	0.571	3.96	< 0.0001***	0.277	0.514	0.29	0.590
Travel	− 0.295	0.555	0.53	0.59	0.250	0.356	0.49	0.483
Forage	0.088	0.539	0.16	0.87	− 0.332	0.347	0.92	0.339
Social	1.992	0.492	4.05	< 0.0001***	0.371	0.177	4.38	0.036*
Tight	0.105	0.394	0.27	0.79	− 0.142	0.528	0.07	0.788
Moderate	0.417	0.542	0.77	0.44	0.122	0.551	0.05	0.825
Spread	0.501	0.491	1.02	0.31	0.541	0.520	1.08	0.298

Parameter	Whistle occurrence model				Whistle frequency model			
	Estimate	Std. error	*z*	*P* value	Estimate	Std. error	Wald	*P* value
Intercept	− 2.006	0.471	4.26	< 0.0001***	− 0.798	0.403	3.92	0.048*
Travel	0.344	0.424	0.81	0.42	− 0.704	0.341	4.28	0.039*
Forage	0.343	0.404	0.85	0.40	0.320	0.475	0.45	0.501
Social	2.795	0.423	6.60	< 0.0001***	0.740	0.260	8.10	0.004**
Tight	− 0.137	0.385	0.36	0.72	0.665	0.240	7.65	0.006**
Moderate	− 0.103	0.313	0.33	0.74	0.441	0.330	1.79	0.184
Spread	0.026	0.274	0.09	0.93	− 0.038	0.188	0.04	0.840

Estimates were averaged across the top two occurrence models where ΔQIC < 4 (Table S2)

Asterisks denote statistical significance (***P < 0.001, **0.001 < *P*< 0.01, *0.01 < *P*< 0.05)

**Table 2:** Parameter estimates for the generalised linear mixed model with binomial family for change in closest male to female as a function of pop rate and whistle rate and first-order alliance as a random effect

	Estimate	Standard error	Confidence interval	*z* value	*P* value
Pop rate	0.56	0.23	(0.10, 1.02)	2.42	0.01*
Whistle rate	0.52	0.43	(− 0.53, 0.99)	1.19	0.23

Estimates were averaged over the top two models where ΔAIC < 4 (Table S3)

Asterisks denote statistical significance (*0.01 < *P*< 0.05)

**Fig. 4:**
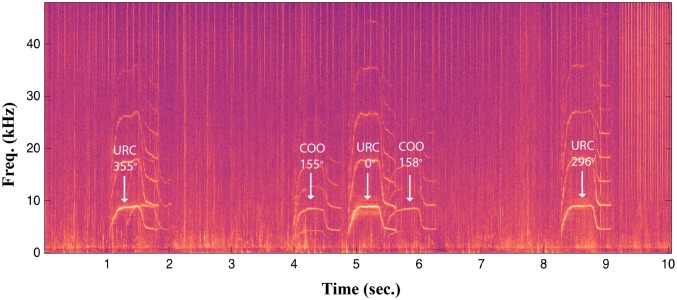
Spectrogram of a non-signature whistle matching sequence between two allied males in Shark Bay (sample rate: 96 kHz, FFT length: 1024, Hanning window function). The three-letter code (animal identification) and the localised bearing (degrees) of the whistle are shown above each whistle

Whistles occurred significantly more often and at higher rates during social interactions, and at lower rates when travelling (Table 1, Fig. 1c). Whistle rates were also higher when group spread was tight compared to widespread (Table 1, Fig. 1d), because dolphins are typically in close proximity when socialising (touching, petting, rubbing, etc.). Thus, proximity only appears indicative of elevated whistle rates due to the socialising behavioural state. Furthermore, whistles were not recorded in five of the 25 follows (Table S1) in which the consorting groups were resting and in close proximity to one another. Being in close proximity and visual contact negates the need for information exchange via the acoustic channel. Signature whistles, i.e. individual identity signals, comprised approximately 28% of all whistles recorded (signature whistles identified in King et al. 2018). This is a conservative estimate as the signature whistles of some of the focal males are yet to be identified. For one first-order alliance in which the signature whistles of all three males were known (King et al. 2018), we observed non-signature whistle matching between two of the males, which immediately preceded a change in behaviour (foraging to travel) by the three males and their female consort (Fig. 4). Finally, when new individuals joined the group (*n* = 58), whistle rates significantly increased but pop rates did not change (Table 3, Fig. 2c, d).

**Table 3:** Parameter estimates for the generalised linear mixed model with binomial family for arrival of new individual(s) as a function of pop rate and whistle rate and first-order alliance as a random effect

	Estimate	Standard error	Confidence interval	*z* value	*P* value
Pop rate	− 0.03	0.09	(− 0.24, 0.12)	0.38	0.69
Whistle rate	0.34	0.11	(0.13, 0.58)	3.13	0.001**

Estimates were averaged over the top two models where ΔAIC < 4 (Table S4)

Asterisks denote statistical significance **0.001 < *P*< 0.01

We note that other vocalisation types, e.g. burst-pulsed calls, were occasionally produced by dolphins in this population, though not at sufficiently high enough rates during these focal follows to include in the analyses. In addition, no other vocalisation types were produced in the context of guard switches.

## Discussion

We show that vocal signals, specifically pop vocalisations rather than the more commonly studied whistles, play an important role in mate guarding behaviour in male bottlenose dolphin alliances. It was previously determined that pops were typically produced by males during consortships, and appeared to function as a female-directed threat vocalisation, inducing her to stay close to the consorting males (Connor and Smolker 1996; Vollmer et al. 2015). Using detailed focal follow data, we found that pops were produced in groups regardless of group spread, suggesting that males continually monitor the location of the female. However, allied male dolphins did significantly increase pop production during guard switches. Acoustic localisation during a subset of guard switching events (*n* = 3) suggests that it is not the nearest male to the female that pops prior to a switch; but males will produce pops whilst approaching the female and current guard (Fig. 3).

The increase in pop production during guard switches can be explained in one of two ways. First, as suggested by previous studies (Connor and Smolker 1996; Vollmer et al. 2015), pop production is directed towards the female only, and any information that males extract from each other’s pop production is a by-product. A male may direct his pops towards the female to check on her location and induce her to move closer, irrespective of whether one of his alliance partners is close to her or not. However, a second possibility, and one that warrants further investigation, is that pops are directed toward both the female and male guard as indicative of the popping males’ motivation to initiate a guard switch. Indeed, on two of the three occasions where pops were localised to the approaching male, the current guard and the female then directly approached the popping male (Fig. 3). One could argue that the female is more likely to bolt in an attempt to escape the alliance in the context of males changing positions, and this alone could explain the significant increase in pop rate. However, we found that pops do not occur at higher rates when the consorting group are widespread from each other in the absence of a guard switch. This suggests that female proximity does not fully explain pop production, but rather it is shaped by a number of contextual and motivational factors. Guard switches may play a role in reducing tension between alliance partners during consortships, a context in which males work together to herd a female, but are also competing for an indivisible fertilisation. Alliance relationships can last for decades and are critical to each male’s reproductive success (Connor and Krützen 2015). As such, maintaining strong social bonds between alliance partners, e.g. sharing access to the female, should take precedence over contesting access to the consorted female. We found no other vocal signal to be consistently associated with guard switches in this study. Future work should use playback experiments to examine sex differences in individual responses to pop trains to determine the intended audience of pops in this context.

Whistle rates increased when new individuals joined the group, consistent with previous work showing that signature whistles are part of a greeting sequence that occurs when free-ranging groups of bottlenose dolphins (*T. truncatus*) encounter one another (Quick and Janik 2012). To date, signature whistles have only been identified for a subset of the males used in this study, thus, specific differences in the use of signature whistles versus non-signature whistles during consortships cannot yet be addressed. Nevertheless, for one first-order alliance with known signature whistles (King et al. 2018), two males participated in a dyadic vocal matching exchange (King and McGregor 2016), with a non-signature whistle type immediately preceding a change in behaviour (Fig. 4). The matching of shared, non-signature whistle types has previously been shown to play a role in coordinated foraging (King and Janik 2015). It is possible that non-signature whistles also play a role in the coordination of behaviour or behavioural changes between allied males, and future work should examine the different roles of signature and non-signature whistles in this population.

Our study presents insight into the role vocal signals play in facilitating behavioural coordination between allied individuals. Bottlenose dolphins (*T. truncatus*) have been shown to understand their partner’s role during a cooperative task (Jaakkola et al. 2018), so they certainly possess the cognitive capacity to negotiate guard switches. Chimpanzees have also demonstrated a clear understanding of their partner’s role during cooperative contexts (Melis et al. 2006). However, while alpha male chimpanzees may tolerate their allies mating with females in exchange for support during conflicts with competitors (Duffy et al. 2007), coercive consortships are always performed by single males. Thus, while both chimpanzees and dolphins have the cognitive skills to utilise their partners as social tools (Seed and Jensen 2011), it is dolphins that actively utilise partners in a cooperative mate guarding context. Our findings shed light on the vocal signals that may facilitate such cooperative behaviour between allied male dolphins.

## Electronic supplementary material

Below is the link to the electronic supplementary material.

Supplementary material 1 (DOCX 21 kb)

Supplementary material 2 (CSV 30 kb)

Supplementary material 3 (CSV 6 kb)

Supplementary material 4 (WAV 1071 kb)
