# Supplementary material for: Vocal behaviour of allied male dolphins during cooperative mate guarding
Source: Anim Cogn. 2019 Jul 17;22(6):991–1000. doi: 10.1007/s10071-019-01290-1 (PMC6834747; doi:10.1007/s10071-019-01290-1)
Supplement: Supplementary file 1 — Supplementary material 1 (DOCX 21 kb) [file 10071_2019_1290_MOESM1_ESM.docx]

**Supplementary Material**

**Shark Bay Ethogram – Behavioural states**

Recorded as the predominant activity that is common to ≥50% of the group for ≥50% of the 5 min. interval.

*Resting:* Dolphins are in a tight group, moving slowly with regular, peduncle or tail-out dives. The group may be moving in a straight line or meandering. During rest there is no evidence of foraging or socializing. Snagging is often observed during resting and resting groups regularly snag en masse. Snagging is when a dolphin floats at the surface and is still or slowly moving. When still the dolphin’s flukes drop to the degree that only the rostral edge of the dorsal fin may show at the surface and the rostrum may be exposed to the top of the mandible. Snagging may last from a few seconds to several minutes.

*Travelling:* Individuals are in parallel orientation (abreast) moving in one general direction (i.e. not varying more than 45 degrees and no rapid changes in direction) for a period of several minutes or during consecutive surfacing bouts.

*Socialising:* Social behaviour is characterised by body contact, rubbing and petting, often accompanied by splashing, surface activity and acoustic behaviour. Bottlenose dolphins also perform a variety of synchronous behaviours.

*Forage:* Foraging often involving lone dolphins or widely dispersed groups. An exception to this occurs when one or more dolphins remain in close proximity to a foraging dolphin for social reasons (i.e., when males are herding a female). Both inter-individual geometry (iig) and dive type are important in determining whether or not dolphins are foraging, independent of observations of feeding. Movement and iig are usually characterised by milling (individuals changing orientation with respect to each other) or meandering (individuals are in parallel orientation repeatedly changing direction) during foraging. In deeper water, foraging usually involves multiple breath surfacing bouts, culminating in a tail-out or peduncle dive. In shallower water, regular bottom grubbing, rapid surfaces and (fish) chases are often observed.

**Table S1.** Recording information for the focal follow data, where second-order alliance membership, IDs of first-order allied males and females are all provided, along with follow duration, and the total number of whistles and pop trains recorded during each follow and used in our analyses. The number of whistles and pop trains localised to the focal group are also provided.

| ID | Recording | Second-order alliance | Focal first-order alliance (female) | Follow Duration (mins) | # Whistles recorded | # Whistles localised | # Pop trains recorded | # Pop trains localised |
| --- | --- | --- | --- | --- | --- | --- | --- | --- |
| 1 | F_S2_2016_05_20 | BW | RAD MIT DAB (CAO) | 40 | 4 | 0 (0%) | 22 | 0 (0%) |
| 2 | F_S10_2016_06_02 | FF | FLP SPL OSM (LUK) | 120 | 79 | 0 (0%) | 41 | 2 (5%) |
| 3 | F2_S10_2016_06_19 | FF | FLP SPL OSM (BAQ) | 155 | 1 | 0 (0%) | 0 | - |
| 4 | F_S2_2016_08_06 | KS | PAS DEE CEB (DUR) | 125 | 159 | 78 (49%) | 86 | 10 (12%) |
| 5 | F_S1_2016_08_07 | RR | COO SMO URC (SOG) | 65 | 50 | 27 (54%) | 11 | 0 (0%) |
| 6 | F_S2_2016_08_14 | KS | PON QUA MOG (SKF) | 120 | 92 | 15 (16%) | 83 | 38 (46%) |
| 7 | F_S2_2016_08_24 | KS | PON QUA PAS (LIC) | 145 | 0 | - | 0 | - |
| 8 | F_S7_2016_08_29 | KS | PON QUA PAS (LIC) | 140 | 7 | 0 (0%) | 0 | - |
| 9 | F_S3_2016_08_26 | RR | COO SMO URC (LIC) | 95 | 148 | 0 (0%) | 12 | 0 (0%) |
| 10 | F_S6_2016_09_04 | RR | COO SMO URC (LIC) | 205 | 64 | 33 (52%) | 256 | 25 (10%) |
| 11 | F_S5_2017_06_14 | KS | DEE IMP MOG (COI) | 310 | 225 | 21 (9%) | 112 | 20 (18%) |
| 12 | F_S10_2017_06_19 | HG | BTS SCM BAG (CLE) | 50 | 0 | - | 0 | - |
| 13 | F_S4_2017_07_12 | HG | BTS VAG NAP (GNY) | 135 | 1 | 1 (100%) | 112 | 84 (75%) |
| 14 | F_S1_2017_07_27 | RR | COO SMO URC (BSS)* | 210 | 349 | 20 (6%) | 342 | 90 (26%) |
| 15 | F_S1_2017_08_03 | RR | COO SMO URC (BSS) | 75 | 34 | 0 (0%) | 0 | - |
| 16 | F_S5_2017_08_07 | HG | SCM SFK BAG (POL) | 120 | 5 | 4 (80%) | 0 | - |
| 17 | F_S4_2017_08_10 | HG | SCM SFK BAG (POL) | 60 | 3 | 2 (67%) | 0 | - |
| 18 | F_S2_2017_08_30 | HG | BTS VAG NAP (DUC) | 370 | 14 | 6 (43%) | 109 | 7 (6%) |
| 19 | F_S3_2017_08_30 | KS | CEB IMP MOG (COI) | 50 | 7 | 2 (29%) | 9 | 0 (0%) |
| 20 | F_S2_2017_09_05 | KS | CEB IMP MOG (COI) | 240 | 14 | 1 (7%) | 82 | 11 (13%) |
| 21 | F_S3_2017_09_08 | BL | SEV PCT SLO GRB (COI) ^†^ | 60 | 0 | - | 7 | 0 (0%) |
| 22 | F_S2_2017_09_23 | PD | BIG RID FRE (DUC) | 75 | 0 | - | 9 | 0 (0%) |
| 23 | F_S1_2017_10_07 | SJ | PAB SIS TAS (HOW) | 60 | 0 | - | 0 | - |
| 24 | F_S1_2017_10_14 | KS/PD**^‡^** | CEB PAS PON (VBE)**^‡^** | 50 | 12 | 0 (0%) | 18 | 0 (0%) |
| 25 | F_S8_2017_10_14 | AR | JUL DEV QID (1AF) | 50 | 0 | - | 22 | 6 (27%) |

***** attempted theft of BSS twice by all five PD.

^†^ two pairs of males (SEV PCT ) and (SLO GRB) tight with COI for duration of the follow.

**^‡^** PD alliance: NAT WAB (RIP) also present.

**Table S2.** Model selection for the binomial Generalized Estimating Equation (GEE) model (occurrence) and Poisson GEE (frequency) for whistle and pop train counts as a function of group activity and group spread categories using the *MuMIn* package in R.

|  | **Pop train occurrence model** | **Pop train frequency model** |
| --- | --- | --- |
| Model | (Intrc) Activity Spread qLik QIC delta weight | (Intrc) Activity Spread qLik QIC delta weight |
| Full  Activity  Spread  Null | -2.34 + + -244 505 0.00 0.744  -2.05 + -248 507 2.13 0.256  -2.10 + -283 581 76.16 0.000  -1.59 -288 584 79.31 0.000 | 0.2765 + + 1638 -3675 0.0 1  0.1398 + 1659 -3651 23.5 0  0.0630 + 1541 -3504 171.1 0  -0.0601 1513 -3415 260.1 0 |
|  | **Whistle occurrence model** | **Whistle frequency model** |
| Model | (Intrc) Activity Spread qLik QIC delta weight | (Intrc) Activity Spread qLik QIC delta weight |
| Full  Activity  Spread  Null | -1.33 + + -270 562 3.45 0.151 -2.13 + -274 558 0.00 0.849  -1.52 + -331 695 136.48 0.000 -1.26 -332 677 119.26 0.000 | -0.798 + + 1886 -3767 0.0 1  -0.167 + 1864 -3724 42.4 0  -0.389 + 1790 -3583 184.1 0  0.219 1712 -3419 347.6 0 |

**Table S3.** Model selection for the generalised linear mixed model with binomial family for change in closest male to female as a function of pop rate and whistle rate with first-order alliance as a random effect using the *MuMIn* package in R.

| Model | (Int) pop rate whistle rate df logLik AIC delta weight |
| --- | --- |
| Full  Pop rate  Whistle rate  Null | -0.655 0.586 3 -124 254 0.00 0.550  -0.708 0.535 0.526 4 -123 255 0.48 0.434  -0.577 0.711 3 -128 262 7.83 0.011  -0.476 2 -130 264 9.47 0.005 |

**Table S4**. Model selection for the generalised linear mixed model with binomial family for arrival of new individual(s) as a function of pop rate and whistle rate with first-order alliance as a random effect using the *MuMIn* package in R.

| Model | (Int) pop rate whistle rate df logLik AIC delta weight |
| --- | --- |
| Whistle rate  Full  Null  Pop rate | -2.61 0.343 3 -183 372 0.00 0.699  -2.60 -0.0356 0.353 4 -183 374 1.84 0.278  -2.57 2 -188 379 7.49 0.017  -2.57 0.0208 3 -188 381 9.42 0.006 |

**Data S1:** Contains the raw data used in the occurrence and frequency GEE models (Table 1), as well as the data for the binomial GEE for group joins vs. vocalisation rates.

**Data S2:** Contains the raw data used for the binomial GLMM for change in nearest male to female vs. vocalisation rates.

**Pops sound file:** wav file of pop train presented in Figure 3a.
